# Phenotypic analysis of antibiotic resistance and genotypic study of the *vacA*, *cagA*, *iceA*, *oipA* and *babA* genotypes of the *Helicobacter pylori* strains isolated from raw milk

**DOI:** 10.1186/s13756-018-0409-y

**Published:** 2018-09-29

**Authors:** Reza Ranjbar, Farid Yadollahi Farsani, Farhad Safarpoor Dehkordi

**Affiliations:** 1 Molecular Biology Research Center, Systems Biology and Poisonings Institute, Baqiyatallah University of Medical Sciences, Tehran, Iran; 2 Molecular Biology Research Center, Systems Biology and Poisonings Institute, Baqiyatallah University of Medical Sciences, Tehran, Iran; 3 Young Researchers and Elites Club, Shahrekord Branch, Islamic Azad University, Shahrekord, Iran

**Keywords:** *Helicobacter pylori*, Raw milk, Antibiotic resistance, Genotyping

## Abstract

**Background:**

Foods with animal origins and particularly milk play a considerable role in transmission of *Helicobacter pylori*. The current study was performed to assess phenotypic characters of antibiotic resistance and genotyping pattern of *vacA*, *cagA*, *iceA*, *oipA* and *babA2* alleles amongst the *H. pylori* strains isolated from raw milk.

**Methods:**

Six-hundred and thirty raw milk samples were collected and cultured on Wilkins Chalgren anaerobe media. Antibiotic resistance and genotyping patterns were studied using disk diffusion and PCR, respectively.

**Results:**

Sixty-seven out of 630 (10.63%) raw milk samples were positive for *H. pylori*. Ovine raw milk (17.27%) samples had the highest prevalence of *H. pylori*, while camel (5.00%) had the lowest. *H. pylori* strains harbored the highest prevalence of resistance against ampicillin (82.08%), tetracycline (76.11%), amoxicillin (74.62%), metronidazole (65.67%) and erythromycin (53.73%). Prevalence of resistance against more than 10 types of antibiotics was 17.91%. *VacA s1a* (83.58%), *m1a* (80.59%), *s2* (77.61%) and *m2* (68.65%), *cagA* (73.13%) and *babA2* (44.77%) were the most commonly detected genotypes. We found that *S1am1a* (56.71%), *s2m1a* (56.71%), *s1 am2* (43.28%) and *s2 m2* (43.28%) were the most commonly detected genotyping pattern. Frequency of *cagA*-, *oipA*- and *babA2*- genotypes were 26.86%, 62.68% and 55.22%, respectively. We found that S1a/cagA+/iceA1/oipA−/babA2- (28.35%), m1a/cagA+/iceA1/oipA−/babA2- (28.35%) and s2/cagA+/iceA1/oipA−/babA2- (26.86%) were the most commonly detected combined genotyping pattern.

**Conclusions:**

Simultaneous presence of *vacA*, *cagA*, *iceA*, *oipA* and *babA2* genotypes in antibiotic resistant *H. pylori* strains indicates important public health issue regarding the consumption of raw milk. However, additional researches are required to find molecular genetic homology and other epidemiological aspects of *H. pylori* in milk.

## Background

Milk of animals provide a package of key nutrients that are difficult to obtain in diets with limited or no dairy products [1]. Dissimilarly, raw milk is not necessarily safe, as evidenced by higher rates of foodborne illnesses associated with its consumption [2–6]. Likewise, there were so many investigations about the considerable prevalence of some specific foodborne pathogens in raw milk [2–6].

*Helicobacter pylori* (*H. pylori*) is a microaerophilic and Gram-negative spiral coccoid flagellated bacterium with 2 to 4 μm in length and 0.5 to 1 μm in width. It is known as one of the main causative agents of duodenal ulcer, peptic ulcer disease, gastric adenocarcinoma, type B gastritis and gastric B-cell lymphoma [7–9]. Human stomach is considered as a main reservoir of *H. pylori* strains [7–9]. In keeping with this, foods with animal origins may play an imperative role in transmission of *H. pylori* infections to human [7–9]. Suitable conditions including pH, activated water (AW), moisture and temperature cause *H. pylori* to easily survive in milk [10]. Raw milk [10], pasteurized milk [7–9] and even sterilized food samples [10] have been introduced as possible emerging sources of *H. pylori* infections. Vacuolating Cytotoxin A (*vacA*) and Cytotoxin Associated Gene A (*cagA*) are two important virulence genes with high importance in the pathogenicity of *H. pylori* infections [7–10]. The *vacA* gene is polymorphic, comprising variable signal regions (type *s1* or *s2*) and mid-regions (type *m1* or *m2*). The *s1* type is additionally divided into *s1a*, *s1b* and *s1c* and the *m1* into *m1a* and *m1b* subtypes. The *cagA* gene has been detected in the severe cases of gastrointestinal disorders and peptic ulcers [7–10]. Induced by contact with the epithelium antigen (*iceA*), outer inflammatory protein (*oip*) and blood group antigen-binding adhesin gene (*babA*) are other important pathogenic genotypes of the *H. pylori* strains [7–11]. Genotyping using these virulence markers is considered as one of the best approaches to study the correlations between *H. pylori* isolates from different samples [11].

Antibiotic therapy is one of the best aspects of treatments for *H. pylori* infections. However, therapeutic options have become somewhat restricted because of the presence of severe resistance in some strains of this bacterium [12]. Documented data disclosed that *H. pylori* strains harbored the high prevalence of resistance against different types of antibiotics [12].

Data on the epidemiology and transmission of *H. pylori* is extremely significant in order to prevent its distribution and to identify high-risk populations, especially in areas that have high rates of infections such as Iran [7–10, 13, 14]. Considering the indistinct epidemiological aspects of *H. pylori* in milk and due to the high prevalence of *H. pylori* all-around the world [7–14], the present investigation was performed in order to study the prevalence rate, genotyping patterns and phenotypic evaluation of antibiotic resistance of the *H. pylori* strains isolated from raw milk samples of bovine, ovine, caprine, buffalo and camel.

## Methods

### Samples

From January to March 2018, total 630 raw milk samples of bovine (*n* = 120), ovine (*n* = 110), caprine (*n* = 130), buffalo (n = 130) and camel (*n* = 140) were arbitrarily collected from the supermarkets of diverse areas of Isfahan province, Iran. All milk samples were collected from traditional dairy farms. Milk samples were kept at refrigerator. Throughout milk collection, the first few squirts were overlooked. The animals which their milk samples collected for this research were clinically healthy, and the milk samples displayed natural physical (color, odor, pH, and density) constancy. Samples (50 ml, in sterile glass bottles) were transported in ice-cooled flasks (at 4 °C) to the laboratory within two hours after collection.

### Isolation of helicobacter pylori

Isolation of *H. pylori* bacteria was performed using the culture technique [7–10, 13, 14]. Twenty-five milliliters of milk sample were used for this purpose. Wilkins Chalgren anaerobe broth (Oxoid Ltd., Basingstoke, UK) was used for this purpose. Microaerophilic conditions (5% oxygen, 85% nitrogen and 10% CO_2_) was prepared using the MART system (MART system, Lichtenvoorde, The Netherland).

### DNA extraction and 16S rRNA-based PCR confirmation

Distinctive colonies of *H. pylori* were additionally approved using the *16S rRNA*-based PCR method. Typical colonies were sub-cultured on Wilkins Chalgren anaerobe broth supplemented with same materials mentioned above [15]. Genomic DNA was then extracted from colonies using a DNA extraction kit (Thermo Fisher Scientific, St. Leon-Rot, Germany). Procedure was performed rendering to the manufacturer’s guidelines. Purity (A260/A280) and concentration of extracted DNA were then checked (NanoDrop, Thermo Scientific, Waltham, MA, USA). The truth of the DNA was assessed on a 2% agarose gel stained with ethidium bromide (0.5 μg/mL) (Thermo Fisher Scientific, St. Leon-Rot, Germany). Polymerase Chain Reaction (PCR) was performed using a PCR thermal cycler (Eppendorf Co., Hamburg, Germany) according to reported procedure [15].

### Study the antibiotic resistance pattern

There are no accepted standardized methods for testing *H. pylori* antimicrobial susceptibilities and the protocols used in this study were based on recently published guidelines [16] and also those of Performance Standards for Antimicrobial Susceptibility Testing- Clinical and Laboratory Standards Institute - NCCLS, 2007 [17]. Briefly, bacterial suspensions were adjusted to the 0.5 McFarland standard (equivalent to 1–2 × 10^8^ cfu/ml) and were used to inoculate Muller Hinton agar plates (Merck, Germany). Antimicrobial disks (ampicillin (10 μg), levofloxacin (5 μg), metronidazole (5 μg), clarithromycin (2 μg), amoxicillin (10 μg), streptomycin (10 μg), cefsulodin (30 μg), erythromycin (5 μg), tetracycline (30 μg), trimethoprim (25 μg), furazolidone (1 μg), rifampin (30 μg), and spiramycin (100 μg) (Oxoid, UK)) were applied and the plates were incubated under microaerophilic conditions at 35 °C for 16–18 h. The zones of growth inhibition produced by each antibiotic were measured and interpreted by standard procedure. Reference strains NCTC 13206 (CCUG 38770) and NCTC 13207 (CCUG 38772) were included as quality controls [18].

### Genotyping analysis

Frequency of *vacA*, *cagA*, *iceA*, *oipA* and *babA* alleles were assessed using PCR [19–22]. Table 1 characterizes the set of primers and PCR circumstances applied for genotyping of *vacA*, *cagA*, *iceA*, *oipA* and *babA* alleles. Initially, all samples were subjected to pre-tests to found suitable time, temperature and volume of reaction. A programmable DNA thermo-cycler (Eppendorf Mastercycler 5330, Eppendorf-Nethel-Hinz GmbH, Hamburg, Germany) was used in all PCR reactions. PCR grade water and *H. pylori* standard strains (SS1, 26,695, Tx30, J99, 88–23 and 84–183) were used as negative and positive controls, respectively. Ten microliters of PCR product were exposed to electrophoresis in a 2% agarose gel in 1X TBE buffer at 80 V for 30 min, stained with SYBR Green. The UVI doc gel documentation systems (Grade GB004, Jencons PLC, London, UK) was applied for analysis of images.

**Table 1:** Set of primers and PCR circumstances applied for genotyping of *vacA*, *cagA*, *iceA*, *oipA* and *babA* alleles

Genes		Primer Sequence (5′-3′)	Size of product (bp)	Volume of PCR reaction (50 μl)	PCR programs
*VacA s*_*1*_ *a*		F: CTCTCGCTTTAGTAGGAGC R: CTGCTTGAATGCGCCAAAC	213	5 μL PCR buffer 10 x 1.5 mM Mgcl_2_ 200 μM dNTP (Thermo Fisher Scientific, St. Leon-Rot, Germany) 0.5 μM of each primers F & R 1.25 U Taq DNA polymerase (Thermo Fisher Scientific, St. Leon-Rot, Germany) 2.5 μL DNA template	1 cycle: 95 °C ------------ 1 min. 32 cycle: 95 °C ------------ 45 s 64 °C ------------ 50 s 72 °C ------------ 70 s 1 cycle: 72 °C ------------ 5 min
*VacA s*_*1*_ *b*		F: AGCGCCATACCGCAAGAG CTGCTTGAATGCGCCAAAC	187		
*VacA s*_*1*_ *c*		F: CTCTCGCTTTAGTGGGGYT R: CTGCTTGAATGCGCCAAAC	213		
*VacA s*_*2*_		F: GCTAACACGCCAAATGATCC R: CTGCTTGAATGCGCCAAAC	199		
*VacA m*_*1*_ *a*		F: GGTCAAAATGCGGTCATGG R: CCATTGGTACCTGTAGAAAC	290		
*VacA m*_*1*_ *b*		F: GGCCCCAATGCAGTCATGGA R: GCTGTTAGTGCCTAAAGAAGCAT	291		
*VacA m*_*2*_		F: GGAGCCCCAGGAAACATTG R: CATAACTAGCGCCTTGCA	352		
*Cag A*		F: GATAACAGCCAAGCTTTTGAGG R: CTGCAAAAGATTGTTTGGCAGA	300	5 μL PCR buffer 10X 2 mM Mgcl_2_ 150 μM dNTP (Thermo Fisher Scientific, St. Leon-Rot, Germany) 0.75 μM of each primers F & R 1.5 U Taq DNA polymerase (Thermo Fisher Scientific, St. Leon-Rot, Germany) 3 μL DNA template	1 cycle: 94 °C ------------ 1 min. 32 cycle: 95 °C ------------ 60 s 56 °C ------------ 60 s 72 °C ------------ 60 s 1 cycle: 72 °C ------------ 10 min
*IceA*	*IceA1*	F: GTGTTTTTAACCAAAGTATC R: CTATAGCCASTYTCTTTGCA	247	5 μL PCR buffer 10 x 2 mM Mgcl_2_ 150 μM dNTP (Thermo Fisher Scientific, St. Leon-Rot, Germany) 0.75 μM of each primers F & R 1.5 U Taq DNA polymerase (Thermo Fisher Scientific, St. Leon-Rot, Germany) 3 μL DNA template	1 cycle: 94 °C ------------ 1 min. 32 cycle: 94 °C ------------ 60 s 56 °C ------------ 60 s 72 °C ------------ 60 s 1 cycle: 72 °C ------------ 10 min
	*IceA2*	F: GTTGGGTATATCACAATTTAT R: TTRCCCTATTTTCTAGTAGGT	229/334		
*OipA*		F: GTTTTTGATGCATGGGATTT R: GTGCATCTCTTATGGCTTT	401	5 μL PCR buffer 10 x 2 mM Mgcl_2_ 150 μM dNTP (Thermo Fisher Scientific, St. Leon-Rot, Germany) 0.75 μM of each primers F & R 1.5 U Taq DNA polymerase (Thermo Fisher Scientific, St. Leon-Rot, Germany) 3 μL DNA template	1 cycle: 94 °C ------------ 1 min. 32 cycle: 94 °C ------------ 60 s 56 °C ------------ 60 s 72 °C ------------ 60 s 1 cycle: 72 °C ------------ 10 min
*BabA*		F: CCAAACGAAACAAAAAGCGT R: GCTTGTGTAAAAGCCGTCGT	105–124	5 μL PCR buffer 10 x 2 mM Mgcl_2_ 150 μM dNTP (Thermo Fisher Scientific, St. Leon-Rot, Germany) 0.75 μM of each primers F & R 1.5 U Taq DNA polymerase (Thermo Fisher Scientific, St. Leon-Rot, Germany) 3 μL DNA template	1 cycle: 94 °C ------------ 1 min. 35 cycle: 94 °C ------------ 60 s 57 °C ------------ 45 s 72 °C ------------ 30 s 1 cycle: 72 °C ------------ 10 min

### Statistical analysis

Data were subjected to Microsoft office Excel (version 15; Microsoft Corp., Redmond, WA, USA). Statistical analysis was performed by means of the SPSS 21.0 statistical software (SPSS Inc., Chicago, IL, USA). Chi-square test and Fisher’s exact two-tailed test were applied to measure any significant relationship. *P* value < 0.05 was considered as statistical significant level.

## Results

Table 2 represents the prevalence of *H. pylori* in different types of raw milk samples. Sixty-seven out of 630 (10.63%) raw milk samples were positive for *H. pylori* strains. All isolates were also approved by the *16SrRNA* gene PCR amplification. Ovine (17.27%) and caprine (13.84%) raw milk samples had the highest prevalence of *H. pylori* strains, while camel (5.00%) had the lowest. Statistically significant difference was seen between type of samples and prevalence of *H. pylori* strains (*P* < 0.05).

**Table 2:** Prevalence of *H. pylori* in different types of raw milk samples

Raw milk samples	No samples collected	*N* (%) of *H. pylori* positive samples	*H. pylori 16SrRNA* PCR confirmation (%)
Bovine	120	9 (7.50)	9 (7.50)
Ovine	110	19 (17.27)	19 (17.27)
Caprine	130	18 (13.84)	18 (13.84)
Buffalo	130	14 (10.76)	14 (10.76)
Camel	140	7 (5.00)	7 (5.00)
Total	630	67 (10.63)	67 (10.63)

**Fig. 1:**
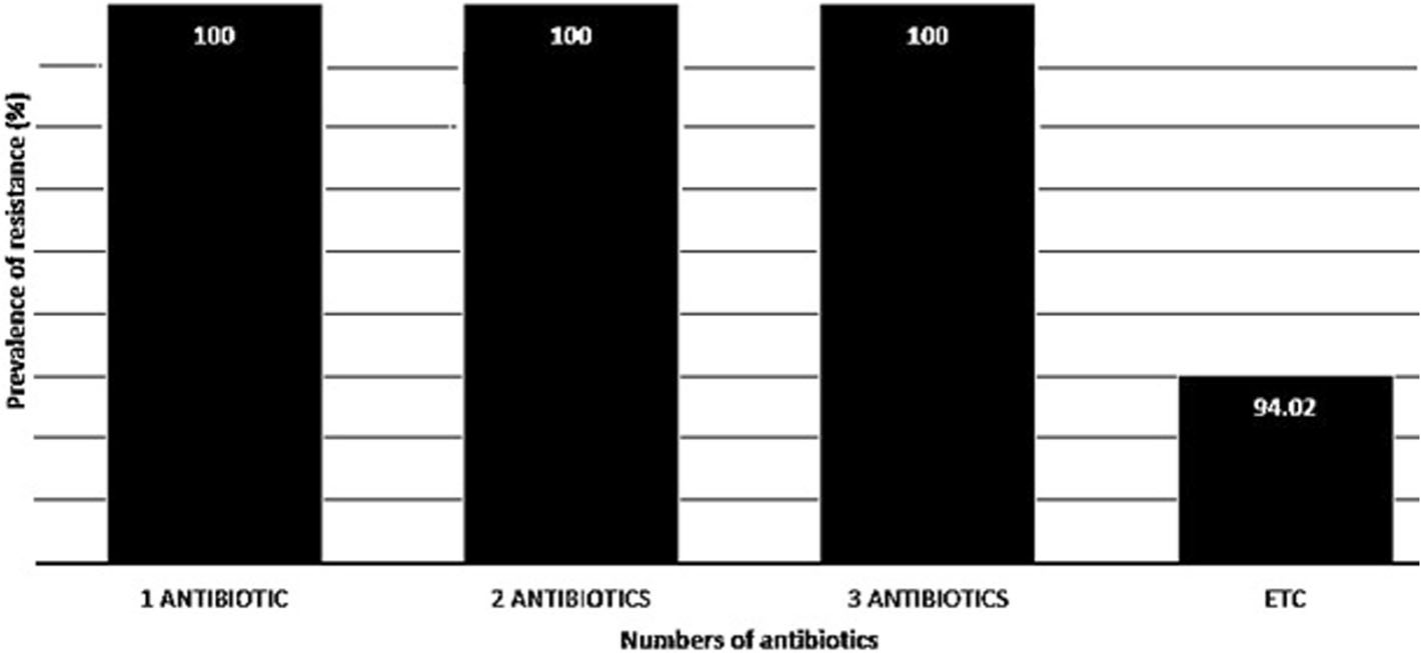
Distribution of multidrug resistant *H. pylori* strains isolated from different types of raw milk. Multidrug resistant *H. pylori* strains were determined as those who had at least simultaneous resistance against 3 or more than 3 types of antibiotics

Table 3 represents the antibiotic resistance pattern of *H. pylori* strains isolated from different types of raw milk samples. *H. pylori* strains harbored the highest prevalence of resistance against ampicillin (82.08%), tetracycline (76.11%), amoxicillin (74.62%), metronidazole (65.67%) and erythromycin (53.73%) antibiotic agents. Furthermore, *H. pylori* strains harbored the lowest prevalence of resistance against cefsulodin (13.43%), furazolidone (13.43%), spiramycin (16.41%) and streptomycin (23.88%). Moreover, prevalence of resistance against clarithromycin, levofloxacin, rifampin and trimethoprim antibiotic agents were 47.76%, 38.80%, 32.83% and 34.32%, respectively. Statistically significant difference was seen between type of samples and prevalence of antibiotic resistance (*P* < 0.05). Figure 1 represents the distribution of multi-drug resistant *H. pylori* strains isolated from different types of raw milk samples. We found that all of the *H. pylori* strains isolated from raw milk samples at least had resistance against 3 different types of antibiotics, while prevalence of resistance against more than 3 types of antibiotics (etc) was 94.02%.

**Table 3:** Antibiotic resistance pattern of *H. pylori* strains isolated from different types of raw milk samples

Type of raw milk samples (N of *H. pylori* strains)	*N* (%) isolates resistant to each antibiotic												
	AM10^a^	Met5	ER5	CLR2	AMX 10	Tet30	Lev5	S10	RIF30	Cef30	TRP25	FZL1	Spi100
Bovine (9)	8 (88.88)	6 (66.66)	4 (44.44)	4 (44.44)	7 (77.77)	7 (77.77)	3 (33.33)	2 (22.22)	3 (33.33)	2 (22.22)	3 (33.33)	2 (22.22)	3 (33.33)
Ovine (19)	18 (94.73)	16 (84.21)	14 (73.68)	13 (68.42)	17 (89.47)	18 (94.73)	12 (63.15)	7 (36.84)	10 (52.63)	4 (21.05)	10 (52.63)	3 (15.78)	3 (15.78)
Caprine (18)	14 (77.77)	12 (66.66)	9 (50)	8 (44.44)	13 (72.22)	13 (72.22)	6 (33.33)	4 (22.22)	5 (27.77)	2 (11.11)	5 (27.77)	3 (16.66)	3 (16.66)
Buffalo (14)	11 (78.57)	8 (57.14)	8 (57.14)	6 (42.85)	10 (71.42)	10 (71.42)	4 (28.57)	2 (14.28)	3 (21.42)	1 (7.14)	4 (28.57)	1 (7.14)	2 (14.28)
Camel (7)	4 (57.14)	2 (28.57)	1 (7.14)	1 (7.14)	3 (42.85)	3 (42.85)	1 (7.14)	1 (7.14)	1 (7.14)	–	1 (7.14)	–	–
Total (67)	55 (82.08)	44 (65.67)	36 (53.73)	32 (47.76)	50 (74.62)	51 (76.11)	26 (38.80)	16 (23.88)	22 (32.83)	9 (13.43)	23 (34.32)	9 (13.43)	11 (16.41)

^a^AM10: ampicillin (10 μg), Met5: metronidazole (5 μg), ER5: erythromycin (5 μg), CLR2: clarithromycin (2 μg), AMX10: amoxicillin (10 μg), Tet30: tetracycline (30 μg), Lev5: levofloxacin (5 μg), S10: streptomycin (10 μg), RIF30: rifampin (30 μg), Cef30: cefsulodin (30 μg), TRP25: trimethoprim (25 μg), FZL1: furazolidone (1 μg) and Spi100: spiramycin (100 μg)

Table 4 represents the distribution of genotypes amongst the *H. pylori* strains isolated from different types of raw milk samples. *VacA s1a* (83.58%), *m1a* (80.59%), *s2* (77.61%) and *m2* (68.65%), *cagA* (73.13%) and *babA2* (44.77%) were the most commonly detected genotypes amongst the *H. pylori* strains isolated from different types of raw milk samples. *VacA s1c* (10.44%), *m1b* (28.35%) and *s1b* (32.83%), *iceA2* (19.40%) and *oipA* (37.31%) had the lowest prevalence amongst the *H. pylori* strains isolated from different types of raw milk samples. Statistically significant difference was seen between type of samples and prevalence of genotypes (*P* < 0.05). Additionally, statistically significant difference was seen between the prevalence of *iceA1* and *iceA2* genotypes (*P* < 0.05).

**Table 4:** Distribution of genotypes amongst the *H. pylori* strains isolated from different types of raw milk samples

Type of raw milk samples (N of *H. pylori* strains)	N (%) isolates harbor each genotype											
	*VacA*							*CagA*	*IceA*		*OipA*	*BabA2*
	*s1a*	*s1b*	*s1c*	*s2*	*m1a*	*m1b*	*m2*		*IceA1*	*IceA2*		
Bovine (9)	7 (77.77)	3 (33.33)	1 (11.11)	6 (66.66)	7 (77.77)	3 (33.33)	6 (66.66)	6 (66.66)	4 (44.44)	2 (22.22)	3 (33.33)	4 (44.44)
Ovine (19)	17 (89.47)	8 (42.10)	2 (10.52)	17 (89.47)	17 (89.47)	7 (36.84)	15 (78.94)	16 (84.21)	10 (52.63)	5 (26.31)	9 (50)	10 (52.63)
Caprine (18)	15 (83.33)	6 (33.33)	2 (11.11)	13 (72.22)	14 (77.77)	5 (27.77)	12 (66.66)	13 (72.22)	8 (44.44)	3 (16.66)	7 (38.88)	8 (44.44)
Buffalo (14)	12 (85.71)	4 (28.57)	1 (7.14)	12 (85.71)	12 (85.71)	3 (21.42)	10 (71.42)	11 (78.57)	6 (42.85)	2 (14.28)	5 (35.71)	6 (42.85)
Camel (7)	5 (71.42)	1 (14.28)	1 (14.28)	4 (57.14)	4 (57.14)	1 (14.28)	3 (42.85)	3 (42.85)	3 (42.85)	1 (14.28)	1 (14.28)	2 (28.57)
Total (67)	56 (83.58)	22 (32.83)	7 (10.44)	52 (77.61)	54 (80.59)	19 (28.35)	46 (68.65)	49 (73.13)	31 (46.26)	13 (19.40)	25 (37.31)	30 (44.77)

Table 5 represents the genotyping pattern of *H. pylori* strains isolated from different types of raw milk samples. *S1am1a* (56.71%), *s2m1a* (56.71%), *s1 am2* (43.28%) and *s2 m2* (43.28%) were the most commonly detected genotyping pattern of the *vacA* alleles of *H. pylori* strains isolated from different types of raw milk samples. Distribution of *cagA*-, *oipA*- and *babA2*- genotypes were 26.86%, 62.68% and 55.22%, respectively. We found that 10.44% of *H. pylori* strains harbored *iceA1*/*iceA2* genotyping pattern. *S1cm1b* (1.49%), *s1 cm2* (4.47%), *s1bm1b* (7.46%), *s1cm1a* (7.46%), s1bm2 (11.94%), *s2m1b* (16.41%) and *s1bm1a* (16.41%) had the lowest prevalence amongst different genotyping patterns of *H. pylori* strains.

**Table 5:** Genotyping pattern of *H. pylori* strains isolated from different types of raw milk samples

Type of raw milk samples (N of *H. pylori* strains)	Genotyping pattern (%)																		
	*s1am1a*	*s1am1b*	*s1 am2*	*s1bm1a*	*s1bm1b*	*s1bm2*	*s1cm1a*	*s1cm1b*	*s1 cm2*	*s2m1a*	*s2m1b*	*s2 m2*	CagA+	CagA-	IceA1/IceA2	OipA+	OipA-	BabA2+	BabA2-
Bovine (9)	5 (55.55)	2 (22.22)	4 (44.44)	2 (22.22)	1 (11.11)	2 (22.22)	1 (11.11)	–	1 (11.11)	4 (44.44)	2 (22.22)	3 (33.33)	6 (66.66)	3 (33.33)	1 (11.11)	3 (33.33)	6 (66.66)	4 (44.44)	5 (55.55)
Ovine (19)	13 (68.42)	4 (21.05)	11 (57.89)	4 (21.05)	2 (10.52)	3 (15.78)	1 (5.26)	1 (5.26)	1 (5.26)	15 (78.94)	5 (26.31)	12 (63.15)	16 (84.21)	3 (15.78)	3 (15.78)	9 (47.36)	10 (52.63)	10 (52.63)	9 (47.36)
Caprine (18)	11 (61.11)	3 (16.66)	10 (55.55)	2 (11.11)	1 (5.55)	2 (11.11)	1 (5.55)	–	1 (5.55)	10 (55.55)	3 (16.66)	8 (44.44)	13 (72.22)	5 (27.77)	2 (11.11)	7 (38.88)	11 (61.11)	8 (44.44)	10 (55.55)
Buffalo (14)	7 (50)	1 (7.14)	3 (21.42)	2 (14.28)	1 (7.14)	1 (7.14)	1 (7.14)	–	–	7 (50)	1 (7.14)	5 (35.71)	11 (78.57)	3 (21.42)	1 (7.14)	5 (35.71)	9 (64.28)	6 (42.85)	8 (57.14)
Camel (7)	2 (28.57)	–	1 (14.28)	1 (14.28)	–	–	1 (14.28)	–	–	2 (28.57)	–	1 (14.28)	3 (42.85)	4 (57.14)	–	1 (14.28)	6 (85.71)	2 (28.57)	5 (71.42)
Total (67)	38 (56.71)	10 (14.92)	29 (43.28)	11 (16.41)	5 (7.46)	8 (11.94)	5 (7.46)	1 (1.49)	3 (4.47)	38 (56.71)	11 (16.41)	29 (43.28)	49 (73.13)	18 (26.86)	7 (10.44)	25 (37.31)	42 (62.68)	30 (44.77)	37 (55.22)

Table 6 represents the combined genotyping pattern of *H. pylori* strains isolated from different types of raw milk samples. We found that s1a/cagA+/iceA1/oipA−/babA2- (28.35%), m1a/cagA+/iceA1/oipA−/babA2- (28.35%), s2/cagA+/iceA1/oipA−/babA2- (26.86%), s1a/cagA+/iceA1/oipA−/babA2+ (25.37%), m1a/cagA+/iceA1/oipA−/babA2+ (25.37%), s2/cagA+/iceA1/oipA−/babA2+ (23.88%), s1a/cagA+/iceA1/oipA+/babA2- (22.38%) and m2/cagA+/iceA1/oipA−/babA2+ (22.38%) were the most commonly detected combined genotyping pattern of *H. pylori* strains isolated from different types of raw milk samples. There were no detected *H. pylori* strains positive for s1b/cagA−/iceA2/oipA+/babA2+, s1c/cagA+/iceA1/oipA+/babA2+, s1c/cagA+/iceA1/oipA+/babA2-, s1c/cagA+/iceA2/oipA+/babA2+, s1c/cagA+/iceA2/oipA+/babA2-, s1c/cagA+/iceA2/oipA−/babA2+, s1c/cagA−/iceA1/oipA+/babA2+, s1c/cagA−/iceA1/oipA+/babA2-, s1c/cagA−/iceA1/oipA−/babA2+, s1c/cagA−/iceA2/oipA+/babA2+, s1c/cagA−/iceA2/oipA+/babA2-, s1c/cagA−/iceA2/oipA−/babA2+, s1c/cagA−/iceA2/oipA−/babA2-, m1b/cagA−/iceA1/oipA+/babA2+ and s1b/cagA−/iceA2/oipA+/babA2+ combined genotyping patterns. Prevalence of s1b/cagA−/iceA1/oipA+/babA2+, s1b/cagA−/iceA1/oipA+/babA2-, s1b/cagA−/iceA2/oipA+/babA2-, s1c/cagA+/iceA1/oipA−/babA2+, s1c/cagA+/iceA2/oipA−/babA2-, s1c/cagA−/iceA1/oipA−/babA2-, s2/cagA−/iceA2/oipA+/babA2+, m1b/cagA−/iceA1/oipA+/babA2-, m1b/cagA−/iceA1/oipA−/babA2+, m1b/cagA−/iceA2/oipA+/babA2- and m2/cagA−/iceA2/oipA+/babA2+ (1.49%) were lower than other detected combined genotyping patterns.

**Table 6:** Combined genotyping pattern of *H. pylori* strains isolated from different types of raw milk samples

Combined genotyping patterns	Distribution^a^ (%)
s1a/cagA+/iceA1/oipA+/babA2+	13 (19.40)
s1a/cagA+/iceA1/oipA+/babA2-	15 (22.38)
s1a/cagA+/iceA1/oipA−/babA2+	17 (25.37)
s1a/cagA+/iceA1/oipA−/babA2-	19 (28.35)
s1a/cagA+/iceA2/oipA+/babA2+	4 (5.97)
s1a/cagA+/iceA2/oipA+/babA2-	5 (7.46)
s1a/cagA+/iceA2/oipA−/babA2+	6 (8.95)
s1a/cagA+/iceA2/oipA−/babA2-	7 (10.44)
s1a/cagA−/iceA1/oipA+/babA2+	6 (8.95)
s1a/cagA−/iceA1/oipA+/babA2-	8 (11.94)
s1a/cagA−/iceA1/oipA−/babA2+	8 (11.94)
s1a/cagA−/iceA1/oipA−/babA2-	10 (14.92)
s1a/cagA−/iceA2/oipA+/babA2+	2 (2.98)
s1a/cagA−/iceA2/oipA+/babA2-	4 (5.97)
s1a/cagA−/iceA2/oipA−/babA2+	5 (7.46)
s1a/cagA−/iceA2/oipA−/babA2-	5 (7.46)
s1b/cagA+/iceA1/oipA+/babA2+	9 (13.43)
s1b/cagA+/iceA1/oipA+/babA2-	11 (16.41)
s1b/cagA+/iceA1/oipA−/babA2+	12 (17.91)
s1b/cagA+/iceA1/oipA−/babA2-	14 (20.89)
s1b/cagA+/iceA2/oipA+/babA2+	2 (2.98)
s1b/cagA+/iceA2/oipA+/babA2-	3 (4.47)
s1b/cagA+/iceA2/oipA−/babA2+	4 (5.97)
s1b/cagA+/iceA2/oipA−/babA2-	5 (7.46)
s1b/cagA−/iceA1/oipA+/babA2+	1 (1.49)
s1b/cagA−/iceA1/oipA+/babA2-	1 (1.49)
s1b/cagA−/iceA1/oipA−/babA2+	2 (2.98)
s1b/cagA−/iceA1/oipA−/babA2-	2 (2.98)
s1b/cagA−/iceA2/oipA+/babA2+	–
s1b/cagA−/iceA2/oipA+/babA2-	1 (1.49)
s1b/cagA−/iceA2/oipA−/babA2+	2 (2.98)
s1b/cagA−/iceA2/oipA−/babA2-	2 (2.98)
s1c/cagA+/iceA1/oipA+/babA2+	–
s1c/cagA+/iceA1/oipA+/babA2-	–
s1c/cagA+/iceA1/oipA−/babA2+	1 (1.49)
s1c/cagA+/iceA1/oipA−/babA2-	2 (2.98)
s1c/cagA+/iceA2/oipA+/babA2+	–
s1c/cagA+/iceA2/oipA+/babA2-	–
s1c/cagA+/iceA2/oipA−/babA2+	–
s1c/cagA+/iceA2/oipA−/babA2-	1 (1.49)
s1c/cagA−/iceA1/oipA+/babA2+	–
s1c/cagA−/iceA1/oipA+/babA2-	–
s1c/cagA−/iceA1/oipA−/babA2+	–
s1c/cagA−/iceA1/oipA−/babA2-	1 (1.49)
s1c/cagA−/iceA2/oipA+/babA2+	–
s1c/cagA−/iceA2/oipA+/babA2-	–
s1c/cagA−/iceA2/oipA−/babA2+	–
s1c/cagA−/iceA2/oipA−/babA2-	–
s2/cagA+/iceA1/oipA+/babA2+	12 (17.91)
s2/cagA+/iceA1/oipA+/babA2-	13 (19.40)
s2/cagA+/iceA1/oipA−/babA2+	16 (23.88)
s2/cagA+/iceA1/oipA−/babA2-	18 (26.86)
s2/cagA+/iceA2/oipA+/babA2+	3 (4.47)
s2/cagA+/iceA2/oipA+/babA2-	4 (5.97)
s2/cagA+/iceA2/oipA−/babA2+	6 (8.95)
s2/cagA+/iceA2/oipA−/babA2-	6 (8.95)
s2/cagA−/iceA1/oipA+/babA2+	5 (7.46)
s2/cagA−/iceA1/oipA+/babA2-	7 (10.44)
s2/cagA−/iceA1/oipA−/babA2+	9 (13.43)
s2/cagA−/iceA1/oipA−/babA2-	10 (14.92)
s2/cagA−/iceA2/oipA+/babA2+	1 (1.49)
s2/cagA−/iceA2/oipA+/babA2-	3 (4.47)
s2/cagA−/iceA2/oipA−/babA2+	4 (5.97)
s2/cagA−/iceA2/oipA−/babA2-	5 (7.46)
m1a/cagA+/iceA1/oipA+/babA2+	12 (17.91)
m1a/cagA+/iceA1/oipA+/babA2-	14 (20.89)
m1a/cagA+/iceA1/oipA−/babA2+	17 (25.37)
m1a/cagA+/iceA1/oipA−/babA2-	19 (28.35)
m1a/cagA+/iceA2/oipA+/babA2+	4 (5.97)
m1a/cagA+/iceA2/oipA+/babA2-	5 (7.46)
m1a/cagA+/iceA2/oipA−/babA2+	6 (8.95)
m1a/cagA+/iceA2/oipA−/babA2-	6 (8.95)
m1a/cagA−/iceA1/oipA+/babA2+	6 (8.95)
m1a/cagA−/iceA1/oipA+/babA2-	7 (10.44)
m1a/cagA−/iceA1/oipA−/babA2+	8 (11.94)
m1a/cagA−/iceA1/oipA−/babA2-	10 (14.92)
m1a/cagA−/iceA2/oipA+/babA2+	2 (2.98)
m1a/cagA−/iceA2/oipA+/babA2-	3 (4.47)
m1a/cagA−/iceA2/oipA−/babA2+	5 (7.46)
m1a/cagA−/iceA2/oipA−/babA2-	5 (7.46)
m1b/cagA+/iceA1/oipA+/babA2+	8 (11.94)
m1b/cagA+/iceA1/oipA+/babA2-	10 (14.92)
m1b/cagA+/iceA1/oipA−/babA2+	10 (14.92)
m1b/cagA+/iceA1/oipA−/babA2-	13 (19.40)
m1b/cagA+/iceA2/oipA+/babA2+	2 (2.98)
m1b/cagA+/iceA2/oipA+/babA2-	3 (4.47)
m1b/cagA+/iceA2/oipA−/babA2+	4 (5.97)
m1b/cagA+/iceA2/oipA−/babA2-	4 (5.97)
m1b/cagA−/iceA1/oipA+/babA2+	–
m1b/cagA−/iceA1/oipA+/babA2-	1 (1.49)
m1b/cagA−/iceA1/oipA−/babA2+	1 (1.49)
m1b/cagA−/iceA1/oipA−/babA2-	2 (2.98)
s1b/cagA−/iceA2/oipA+/babA2+	–
m1b/cagA−/iceA2/oipA+/babA2-	1 (1.49)
m1b/cagA−/iceA2/oipA−/babA2+	2 (2.98)
m1b/cagA−/iceA2/oipA−/babA2-	2 (2.98)
m2/cagA+/iceA1/oipA+/babA2+	11 (16.41)
m2/cagA+/iceA1/oipA+/babA2-	13 (19.40)
m2/cagA+/iceA1/oipA−/babA2+	15 (22.38)
m2/cagA+/iceA1/oipA−/babA2-	18 (26.86)
m2/cagA+/iceA2/oipA+/babA2+	2 (2.98)
m2/cagA+/iceA2/oipA+/babA2-	4 (5.97)
m2/cagA+/iceA2/oipA−/babA2+	5 (7.46)
m2/cagA+/iceA2/oipA−/babA2-	6 (8.95)
m2/cagA−/iceA1/oipA+/babA2+	3 (4.47)
m2/cagA−/iceA1/oipA+/babA2-	6 (8.95)
m2/cagA−/iceA1/oipA−/babA2+	8 (11.94)
m2/cagA−/iceA1/oipA−/babA2-	10 (14.92)
m2/cagA−/iceA2/oipA+/babA2+	1 (1.49)
m2/cagA−/iceA2/oipA+/babA2-	3 (4.47)
m2/cagA−/iceA2/oipA−/babA2+	3 (4.47)
m2/cagA−/iceA2/oipA−/babA2-	5 (7.46)

^a^Distribution was achieved based on the total numbers of 67 *H. pylori* isolates

## Discussion

*H. pylori* is a common bacterium with high microbiological and clinical importance and about 50% of the world’s population, depending to the geographic location considered, has been estimated to have been infected with this organism. Despite the high incidence of the infection, the reservoir for *H. pylori* and the routes of infection are still indeterminate and various routes of transmission have been recommended [23]. Moreover, epidemiological investigations suggest that transmission of *H. pylori* between individuals happens both via the oral–oral and fecal–oral routes [23]. In keeping with this, fecal–oral transmission has more significant implications than since *H. pylori* may occur in food and water supplies subsequent to fecal contamination [24]. Besides, the isolation of *H. pylori* in drinking water [13, 14], raw vegetables [7, 9], salads [7, 9], meat [25, 26], ready to eat foods [27, 28], sterilized foods [29, 30] and foods with animal origin such as milk [31–35], suggests that these foods may act as vehicles for transmission of *H. pylori* to human population.

The present study was performed to assess the prevalence rate, genotyping patterns and antibiotic resistance properties of *H. pylori* strains isolated from different types of raw milk samples. Totally, 10.63% of raw milk samples were positive for *H. pylori* strains. Prevalence of *H. pylori* strains in raw milk samples of bovine, ovine, caprine, buffalo and camel were 7.50, 17.27, 13.84, 10.76 and 5.00%, respectively. Several studies have been conducted in this field. Talaei et al. (2015) [36] reported that the total prevalence of *H. pylori* strains amongst the cow, sheep, goat and buffalo milk samples were 16.00, 13.79, 4.76, 13.33 and 20.00%, respectively. Quaglia et al. (2008) [35] determined that the prevalence of *H. pylori* strains in sheep, cow and goat milk samples were 33.00%, 50.00% and 25.60%, respectively. Mousavi et al. (2014) [10] described that the prevalence of *H. pylori* strains in bovine, ovine, caprine, buffalo and camel milk samples were 16.66, 35.00, 28.00, 15.00 and 13.30%, respectively. Rahimi and Kheirabadi (2012) [37] noted that the prevalence of *H. pylori* strains in raw bovine, ovine, caprine, buffalo and camel milk samples were 1.41, 12.20, 8.70, 23.40 and 3.60%, respectively. Osman et al. (2015) [38] revealed that the prevalence of *H. pylori* in raw milk samples of different parts of Sudan had a range of 7 to 38%. Similar results have been reported for the high prevalence of *H. pylori* in milk samples from Japan (72.20%) [32], Greece (20.00%) [34], Italy 1.80%) [39] and Iran (16.00%) [40].

Foods presenting intrinsic factors, including water activity higher than 0.97 and pH ranging from 4.9 to 6.0 such as raw milk, theoretically could provide conditions for survival of *H. pylori* [7–9]. Therefore, it is not surprising that the *H. pylori* strains has the high prevalence in raw milk samples of our investigation. High prevalence rate of *H. pylori* in milk samples of our research is may be due to the low levels of hygienic conditions of milking procedure. Furthermore, considering the boost prevalence of *H. pylori* in healthy human carrier, contamination due to poor hygiene management of open package of milk, has more important implications for the transmission of the infection through foods. Milk, that could become contaminated during production or because of low hygiene after the open of package, is considered to be one of the most likely vehicles for infection [31, 32]. Insufficient post-processing hygienic management of the milk, can carry the contamination of the matrix by humans. Despite of the low prevalence of *H. pylori* strains in some kinds of studied milk samples, the infectious dose of *H. pylori* is presumably low [31, 32]. Therefore, it is an important public health threat regarding the consumption of raw milk. The urea-dependent acid resistance of *H. pylori* may account for the long-term survival of *H. pylori* in an acidic environment including raw milk [29]. Higher prevalence of *H. pylori* in raw ovine milk samples is may be due to the more suitable conditions present in ovine milk such as higher fat, protein and water activity and also optimum pH. Furthermore, ovine milk may have a higher qualification for growth and survival of *H. pylori* strains. Moreover, differences in the feed of ovine with bovine, buffalo, camel and even caprine species may affect the prevalence rate of bacteria presented in their milk. Higher prevalence of *H. pylori* in raw ovine milk was also reported by previous investigations [10, 31, 32, 34–43]. Using thorns and thistles in deserts and living away from humans and the polluted environment of cities are the most important probable reasons for the lower prevalence of *H. pylori* in camel milk. Lower prevalence of *H. pylori* in raw camel milk was also reported by previous investigations [10, 37, 44, 45].

We described that *H. pylori* bacteria exhibited the maximum prevalence of resistance against ampicillin, tetracycline, amoxicillin, metronidazole and erythromycin antibiotics. Boost prevalence of resistance against human-based antibiotics such as metronidazole, erythromycin, clarithromycin, levofloxacin, amoxicillin, streptomycin, rifampin, cefsulodin, trimethoprim, furazolidone and spiramycin in *H. pylori* bacteria isolated from raw milk samples characterized their anthropogenic origin. Reversely, boost prevalence of resistance against animal-based antibiotics such as ampicillin and tetracycline in *H. pylori* bacteria isolated from raw milk samples characterized their animal origin. As it displayed, majority of *H. pylori* bacteria exhibited resistance against human-based antibiotics. Extreme, illegal and prohibited prescription of antibiotics in medicine and also veterinary caused momentous surge in antibiotic resistance. Frequent researches have been accomplished, globally. Among plentiful examines performed on the antibiotic resistance of *H. pylori* bacteria, discoveries of Hemmatinezhad et al. (2016) [46] (amoxicillin (94.59%), ampicillin (93.24%), metronidazole (89.18%), tetracycline (72.97%) and erythromycin (58.10%)), Yahaghi et al. (2014) [9] (metronidazole (77.96%), amoxicillin (67.79%), ampicillin (61.01%), and erythromycin (23.72%)) and Mousavi et al. (2014) [10] (ampicillin (84.4%), tetracycline (76.6%), erythromycin (70.5%), metronidazole (70%), and clarithromycin (17.70%)) were similar to our findings. Clinical investigations conducted in Iran, China, India, Nigeria, Taiwan, Senegal, Thailand, Saudi Arabia, Brazil, Egypt, Argentina and Colombia disclosed that *H. pylori* bacteria of human clinical specimens displayed boost prevalence of resistance against aminoglycosides, tetracyclines, penicillins, macrolides and metronidazole [47] which was parallel to our results.

We also found that *vacA s1a*, *s2*, *m1a* and *m2*, *cagA*, *iceA1*, *oipA* and *babA2* genotypes, *s1am1a*, *s2m1a*, *s1 am2*, *s2 m2*, *cagA*-, *oipA*- and *babA2*- patterns and s1a/cagA+/iceA1/oipA−/babA2-, m1a/cagA+/iceA1/oipA−/babA2-, s2/cagA+/iceA1/oipA−/babA2-, s1a/cagA+/iceA1/oipA−/babA2+, m1a/cagA+/iceA1/oipA−/babA2+, s2/cagA+/iceA1/oipA−/babA2+, s1a/cagA+/iceA1/oipA+/babA2- and m2/cagA+/iceA1/oipA−/babA2+ combined genotyping patterns were the most commonly detected virulence characters of *H. pylori* strains isolated from raw milk samples. High prevalence of *vacA*, *cagA*, *iceA1*, *oipA* and *babA2* genotypes was also reported in the *H. pylori* strains isolated from clinical samples of human and animal species [48–51]. Furthermore, high prevalence of these genotypes has been reported in the *H. pylori* strains isolated from different types of food samples [8–10, 14, 36, 42–45, 52]. Adjacent association of *vacA*, *cagA*, *iceA*, *oipA* and *babA2* genotypes of *H. pylori* bacteria with secretion of interleukin-8 and cytotoxin, adhesion to gastric epithelial cells, occurrence of inflammatory effect, vacuolization, apoptosis procedure in gastric epithelial cells, peptic ulceration, increase acute neutrophilic infiltration, interleukin-10 secretion and inflammation, has been presented previously [48–50]. Since *H. pylori* isolates in our investigation harbored *vacA*, *cagA*, *iceA*, *oipA* and *babA2* genotypes, therefore consumption of raw milk contaminated with virulent strains of *H. pylori* may aggravate duodenal ulceration, gastric mucosal atrophy and gastric cancer. Additionally, some of *H. pylori* isolates were simultaneously positive for more than one detected genotypes which poses their higher pathogenicity. Similar genotyping patterns of *H. pylori* strains recovered from human clinical samples were also reported previously [53–56].

A possible relationship between virulence factors and antimicrobial resistance has been suggested. A study conducted in 2009 in Ireland reported that the absence of *cagA* may be a risk factor for developing metronidazole resistance [57]. Other studies have found an association between clarithromycin resistance mutations and the less virulent *vacA* genotypes [58]. Another report revealed that *cagE* and *vacA* S1 correlated with clarithromycin and metronidazole resistance [59], while others found that neither *cagA* nor *vacA* was associated with resistance [60, 61]. Therefore, it is important to found any significant relationship between the presence of virulence markers and antibiotic resistance amongst the *H. pylori* strains.

Triple therapy, including two antibiotics, amoxicillin and clarithromycin, and a proton pump inhibitor given for a week has been recommended as the treatment of choice at several consensus conferences [62]. However, this treatment may fail for several reasons, as reported elsewhere [63]. In fact, the main reason for failure was found to be *H pylori* resistance to one of the antibiotics used (that is, clarithromycin). Other treatments have also been proposed, including metronidazole, a drug for which resistance is also a problem although to a lesser extent, as well as tetracycline, fluoroquinolones, and rifamycins for which resistance has become an emerging issue [64]. Results of the present investigation showed that application of furazolidone, streptomycin and cefsulodin may be effective for treatment of the cases of *H. pylori* infections. Reduction in the antibiotic prescription and also prescription of antibiotics according to the results of the disk diffusion can reduce the risk of antibiotic resistance. Using medicinal plants and especially those with high antimicrobial effects is a practical alternative way for treatment of *H. pylori* infection.

## Conclusions

To put it in a nutshell, we recognized a great numbers of virulent and resistant *H. pylori* bacteria in raw milk samples of bovine, ovine, caprine, buffalo and camel species. Boost incidence of *H. pylori* bacteria in raw milk characterizes that these samples may be the natural reservoirs of the bacteria and can spread *H. pylori* to human. Moreover, some of the *H. pylori* bacteria of our research harbored *vacA*, *cagA*, *iceA*, *oipA* and *babA2* genotypes together which represents the high pathogenicity. Furthermore, higher prevalence of *iceA1*+ strains than *iceA2*+, *oipA*- than *oipA*+ and finally *babA2*- than *babA2*+ is another important finding of our study. Additionally, presence of 97 diverse combined genotyping patterns with high distribution of s1a/cagA+/iceA1/oipA−/babA2-, m1a/cagA+/iceA1/oipA−/babA2-, s2/cagA+/iceA1/oipA−/babA2-, s1a/cagA+/iceA1/oipA−/babA2+, m1a/cagA+/iceA1/oipA−/babA2+, s2/cagA+/iceA1/oipA−/babA2+, s1a/cagA+/iceA1/oipA+/babA2- and m2/cagA+/iceA1/oipA−/babA2+ is another interesting finding of our research. Similarities in the genotyping pattern of *H. pylori* strains between various milk sources represent their same route of infection. High prevalence of multi-drug resistant *H. pylori* strains shows that raw milk of bovine, ovine, caprine, buffalo and camel species may be reservoir of antibiotic resistant *H. pylori*. Prescription of cefsulodin, furazolidone, spiramycin and streptomycin may be effectual for treatment of cases of *H. pylori* infections due to the consumption of raw milk. Additional researches are essential to recognize the rates of the molecular genetic homology of *H. pylori* bacteria isolated from milk and dairy samples and those of human clinical specimens to confirm the zoonotic aspects of *H. pylori*.

## Abbreviations

BabA: Blood group Antigen-Binding Adhesin gene
CagA: Cytotoxin Associated Gene A IceA Induced by Contact with the Epithelium Antigen
*H. pylori*: Helicobacter pylori
*Oip*: Outer Inflammatory Protein
PCR: Polymerase Chain Reaction
SPSS: Statistical Package for the Social Sciences
VacA: Vacuolating Cytotoxin A
